# Using participatory epidemiology to assess factors contributing to common enteric pathogens in Ontario: results from a workshop held at the Ontario Veterinary College, University of Guelph, Ontario

**DOI:** 10.1186/1471-2458-14-405

**Published:** 2014-04-27

**Authors:** Shannon A Harding, E Jane Parmley, Karen E Morrison

**Affiliations:** 1 Department of Population Medicine, University of Guelph, 50 Stone Road East, Guelph, Ontario N1G 2 W1, Canada; 2 Public Health Agency of Canada, 160 Research Lane, Suite 103, Guelph, Ontario N1G 5B2, Canada

**Keywords:** Participatory epidemiology, Enteric pathogens, Most likely source of infection, Contributing factors

## Abstract

**Background:**

Common enteric pathogens that cause gastrointestinal illness are transmitted to humans through food, water or direct contact. This poses a significant concern to public health as enteric pathogens can cause disease in a large number of people, and cost a substantial amount to treat and prevent. In order to gain a better understanding of the occurrence of enteric disease in Ontario, this study explored public health professionals’ perceptions of major contributing factors for common enteric pathogens.

**Methods:**

A case study was conducted as part of a two week training workshop in Participatory Epidemiology held at the Ontario Veterinary College, University of Guelph, in May 2013. Eight semi-structured interviews and four focus groups were conducted with representatives from the Public Health Agency of Canada, the University of Guelph, and three health regions in Southern Ontario. Written notes and pictures captured the qualitative information provided. Results were then analyzed using the mixed methods techniques of triangulation, convergence, and paradox.

**Results:**

A total of fifty factors that contribute to enteric disease were identified across all interviews and focus groups. These contributing factors were grouped into key themes (travel, food handling, industry (farm-to-fork), water, geography, demographics, and behaviours) and were categorized as either a risk factor or susceptibility factor. Informants emphasized the complex relationships between the identified factors, and highlighted why these complexities make it difficult to determine where and how a person most likely acquired an enteric pathogen. Workshop participants observed differences in the type and quality of information collected during interviews and focus groups; we hypothesize that this may be attributed to the dynamics between group members (i.e. focus group discussions) as opposed to one-on-one interviews.

**Conclusions:**

The information gathered will serve as a starting point to further explore contributing factors for common enteric pathogens. The identified complexities would be best explored by conducting additional surveillance, as well as interviews and focus groups with a more diverse group of stakeholders. This type of qualitative study can enhance knowledge of enteric pathogen surveillance and contribute to the development of resources and initiatives to holistically address the occurrence of gastrointestinal illness.

## Background

Infectious enteric disease is caused by the ingestion of bacteria, parasites, or viruses [1]. These pathogens are transmitted from contaminated food and water, or by direct contact between infected animals and people [2]. Upon infection with an enteric pathogen, individuals may present with gastro-intestinal related symptoms, including nausea, vomiting, and diarrhea [1]. Gastro-intestinal illness affects a large number of people and while many cases are mild and self-limiting, more severe forms of disease can lead to serious chronic health problems. Consequently, these diseases have a high economic and social cost in lost days of work and treatment, particularly for long term sequelae [2]. In Ontario, there are an estimated 1.3 cases of acute gastrointestinal illness (AGI) per person-year [1]; nationally, it is estimated that AGI costs the Canadian economy $115 CAD per person annually [3]. Currently, there are many knowledge gaps regarding the various factors (e.g. geographic location and individual behaviours) that contribute to the transmission of enteric pathogens and the severity of disease.

Attributing the source of infection for many of these pathogens can be difficult. For example, the exposure source is unknown in over 50% of the common enteric pathogen cases in Ontario [4]. These unidentified exposures could include contaminated food or water, food safety practices, unpasteurized milk, occupation, environment, and animal-to-person or person-to-person contact [5]. Furthermore, incubation periods (the time between exposure and onset of disease symptoms) make it hard to remember past meals and behaviours. In Ontario, it is estimated that for each recorded case of enteric disease, there are 313 unreported cases [6]. Under-reporting is often due to individuals failing to seek medical attention, which may be caused by mild symptoms or embarrassment to submit a stool sample [7]. The high level of under-reporting contributes to skewed (biased) perceptions of common factors contributing to enteric disease, as the reported cases may be different from the majority of cases in a community.

Across Canada, surveillance systems are in place to aid in the determination of these contributing factors. One example is Canada’s National Integrated Enteric Pathogen Surveillance Program (FoodNet Canada), a sentinel site surveillance program that collects data about enteric pathogens [2]. One of their goals is to determine how pathogens are transmitted and how they infect an individual [5]. Within a sentinel site, FoodNet Canada collaborates with local health units that investigate suspect and confirmed cases of enteric disease. The public health inspectors attempt to identify the most likely source of infection (MLSI) through a set of standardized questionnaires administered to cases [5]. To identify the MLSI, inspectors use their unique perspective and professional experience to synthesize information obtained in interviews and to make an educated guess about where an infection originated.

The goal of the present study was to gain a better understanding of the various factors that contribute to the occurrence of common enteric pathogens in Southern Ontario and how these factors may be influenced by geography (location), culture, demographics, individual behaviours, and future trends. These contributing factors were explored using participatory epidemiology (PE) techniques. PE is an emerging field in public health that is based on traditional epidemiological concepts and allows for the exploration of interactions between the host, agent, and environment within a more social context. The PE methodology is flexible, inexpensive, and employs a variety of techniques such as interviewing, mapping, and ranking to study disease patterns within a population and to identify pertinent information gaps [8]. PE has been predominantly applied in a developing world context and a contribution of this study was to apply the techniques in a developed world context to answer three main research questions:

1. What factors influence how public health practitioners determine the most likely source of infection?

2. Does the perceived importance of these contributing factors change with location, or with the individual characteristics of the affected people?

3. How does the enteric disease incidence and distribution of contributing factors vary across individual health regions and between different health regions?

## Methods

Workshop participants conducted 8 one-hour semi-structured interviews and 4 two-hour focus groups which were carried out as training exercises in a PE workshop case study held May 6 to May 17, 2013 at the Ontario Veterinary College, University of Guelph, Ontario. The workshop participants were a diverse group of graduate students (n = 10), public health professionals (n = 3), and professors (n = 3) of varying age, gender, and experience. The volunteer stakeholders included public health professionals employed by the Public Health Agency of Canada (PHAC), the University of Guelph, and past or present public health inspectors from three health regions in Southern Ontario. Workshop coordinators electronically mailed invitation letters to prospective volunteer stakeholders prior to the start of the workshop. At the time of the interview or focus group, each stakeholder was provided with an information sheet for informants that included details about the study purpose and procedures, potential risks and benefits, confidentiality, rights of participants and contact information. Ethical clearance was not required from the Research Ethics Board at the University of Guelph as the study focus was to ask informants their professional opinion. To maintain confidentiality, the names of stakeholders and their affiliated organization or health region were omitted.

The participating health regions were identified as health region A, B, and C. Health region A was a mix of urban and rural communities with both traditional and progressive agricultural practices; B was very urban and has a large South Asian immigrant population; and C was primarily rural with progressive agricultural practices.

Each semi-structured interview was conducted by two workshop participants with one public health inspector either at the health region office or at one of the PHAC offices in Guelph. The purpose of the interviews was to explore 1) why enteric disease incidence varies between and within health regions, 2) what influences an investigator’s perceptions of the MLSI, and 3) how their perceptions have changed over the course of their career. Prior to the interviews, a set of preliminary questions was developed by the workshop coordinators and shared with public health inspectors and their managers. These questions were intended to directly inform the 3 main research questions outlined previously. For example: In your opinion, why does enteric disease vary across Ontario? This is not typical of semi-structured interviews but was done in response to requests from participating health regions, time constraints, and the need to collect consistent information across all interviews.

Each focus group discussion was led by 3 to 5 workshop participants with a small group of 4 to 7 PHAC and/or University of Guelph employees at one of the PHAC offices in Guelph. Four main themes were pre-identified for the discussion groups: demographics, geography (location), culture and ethnicity, and future trends. Each group was asked to specifically report on one theme to answer the question ‘How does theme ‘*x’* affect exposure routes of important enteric pathogens in Ontario?’ However, any and all themes or topics identified in the focus groups were recorded and discussed.

**Figure 1:**
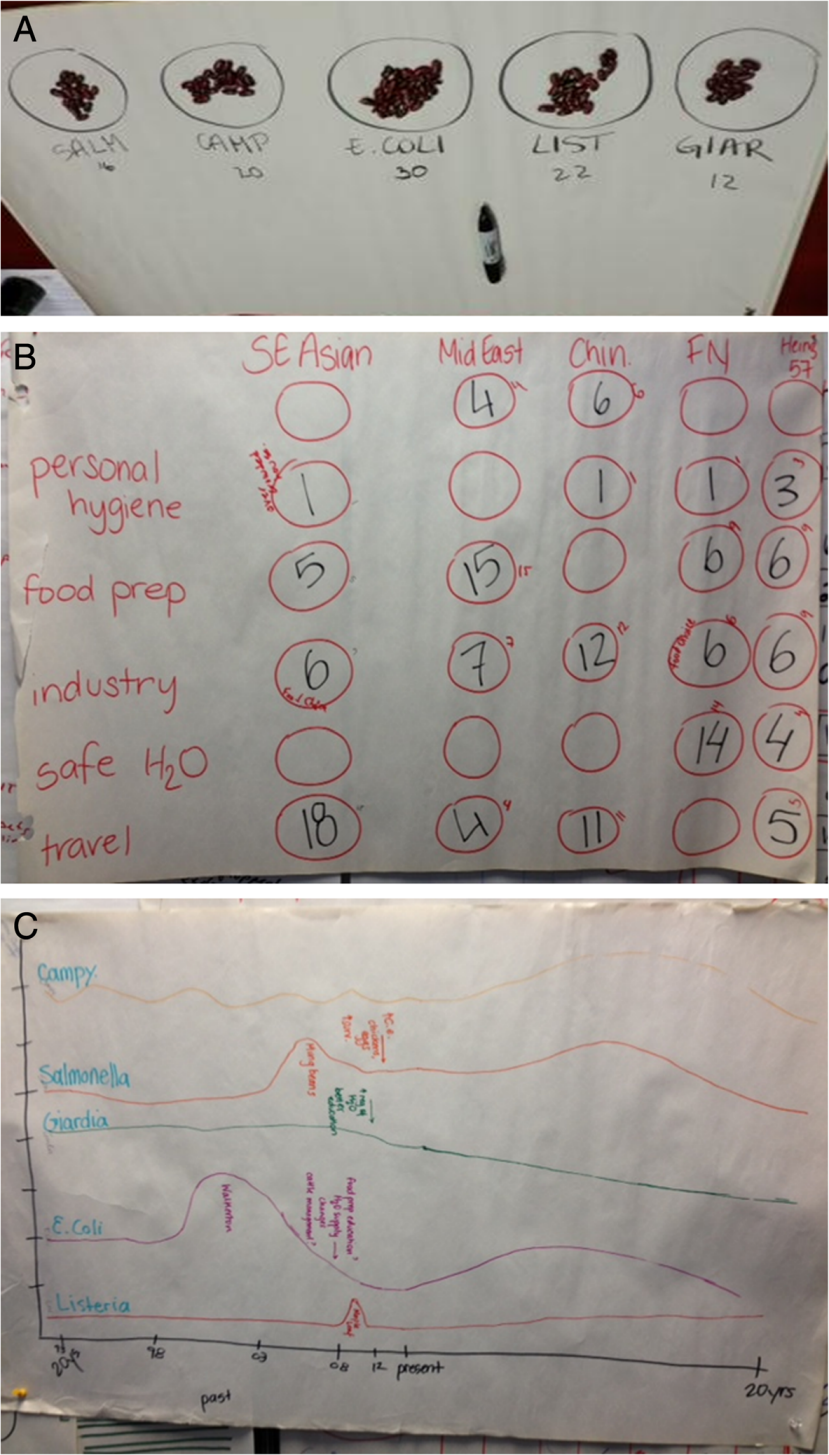
**Participatory epidemiology tools and techniques. A**. This is an example of a proportional piling activity completed during a focus group. Informants were asked to identify the degree of importance for identified enteric pathogens using 100 beans as counters. Informants attributed 16, 20, 30, 22, and 12 beans to S*almonella*, *Campylobacter*, *E. coli*, Listeria, and *Giardia* respectively. A higher bean count represents a pathogen that was considered more important than another. In this example, *E. coli* was considered the most important. **B**. This is an example of a matrix activity completed during a focus group. Informants were asked to attribute beans to identify contributing factors associated with different ethnicities. Ethnicities (located at top of columns) included South (SE) Asian, Middle Eastern, Chinese (Chin), First Nations (FN), and mixed ethnicities (Heinz 57). Factors (located at left of rows) included personal hygiene, food preparation, industry, safe water, and travel. A higher bean count represents a contributing factor or key theme that was considered as the most concerning for a particular ethnicity. In this example, the most concerning factor for South Asian was travel, for Middle Eastern was food preparation, for Chinese was industry (closely followed by travel), for First Nations was safe water, and for mixed ethnicity was food preparation and industry (closely followed by travel and safe water). **C**. This is an example of a timeline activity completed during a focus group. Informants were asked to draw past, present, and future incidence trends for identified important enteric pathogens. From top to bottom, trend lines were predicted for *Campylobacter, Salmonella, Giardia, E. coli,* and Listeria respectively.

Various PE techniques were used during interviews and focus groups. Proportional piling allowed relative scores to be assigned to various categories related to one criterion using designated counters (e.g. 100 beans) [8]. For example, informants were asked to identify a list of pathogens and then assign them scores according to their importance (Figure 1A). Matrices were also used; these are a series of proportional piling exercises where a list of items is scored against a number of indicators to generate a grid [8]. For example, some focus groups were asked to complete a matrix comparing lists of previously identified enteric pathogens and contributing factors (indicators) (Figure 1B). Simple ranking activities were also used to create ordered lists of diseases and factors according to their importance [8]. Finally, some informants were asked to develop timelines to illustrate when disease incidence peaked and fell along a time continuum (past, present, and future) using historical outbreaks and events as a guide/reference to the year. All data and observations from these PE techniques were collected using written notes and photographs for visual representation.

The qualitative data/observations obtained from different sources (e.g. interview and focus group notes, existing case reports) were analyzed using the mixed methods technique of triangulation, convergence and paradox [9]. Triangulation was used as a method to crosscheck research findings with other sources of information, including secondary data sources. The contributing factors Identified by informants were grouped into key themes and then the key themes were grouped into major categories. Each theme was coded to determine how often it was discussed and to what depth. The four classifications used to code were: 1) strongly discussed, 2) mentioned but not explored, 3) not covered or hardly discussed, and 4) probed but received no significant response. The identified themes and codes were agreed upon by all workshop participants and coordinators. The findings were further analysed to determine which key themes were most commonly identified and discussed across interviews and focus groups (convergence) and where the informants disagreed or identified different contributing factors (paradox). Additional data analysis was done to calculate average ranks and scores to determine informants’ perceptions of the most important pathogens. The qualitative results reported were assessed to ensure adherence to qualitative research review guidelines; a RATS checklist was completed for Relevance of the study question, Appropriateness of qualitative method, Transparency of procedures and Soundness of interpretive approach (Additional file 1).

**Figure 2:**
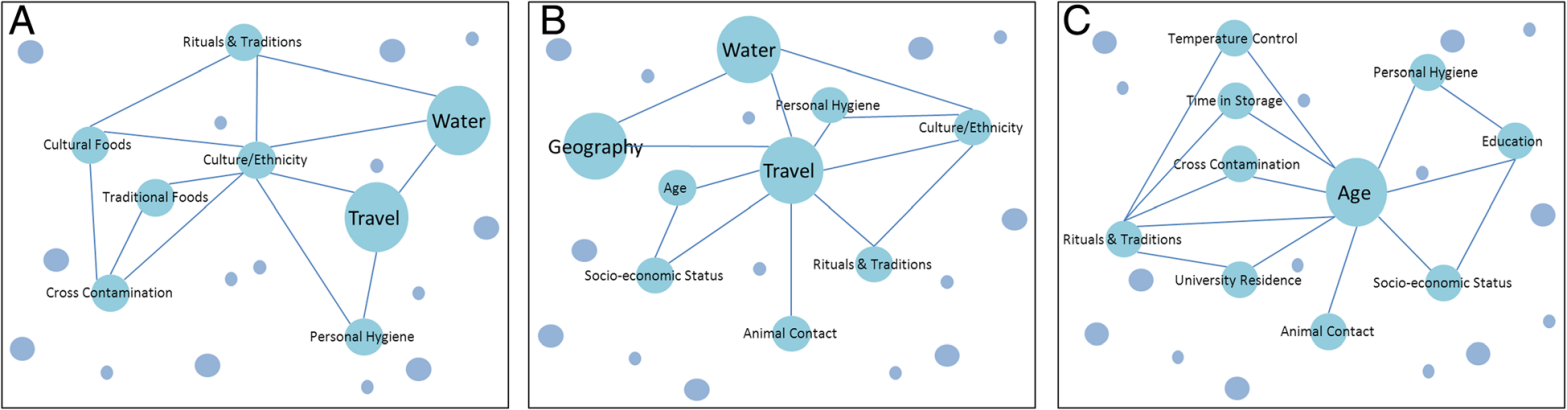
**Contributing factor complexities.** Many informants identified various associations between contributing factors (risk and susceptibility factors) and key themes. To illustrate these associations, factors were mapped analogous to constellations in the night sky. Large circles represent key themes and small circles represent contributing factors. The associations identified by informants were grouped to form constellations (outlined by connecting lines). The 3 diagrams exemplify some of the main complexities and associations discussed during the case study. These included risk and susceptibility factors associated with: **A**. culture and ethnicity, **B**. travel, and **C**. age.

Notes about interview and focus group findings as well as class discussions were reviewed and synthesized to identify examples of important relationships between the factors contributing to enteric disease. To illustrate the complexity we selected three factors (age, travel and culture/ethnicity) for which informants had highlighted complexity and important connections with other contributing factors or key themes. We present the selected factors and their connections analogous to constellations in the night sky: the larger circles represent key themes and the smaller circles represent contributing factors within those themes (Figure 2).

## Results

The informants that agreed to participate represented a small subset from each of the health regions, academic groups and government groups that were approached by workshop coordinators. Not everyone who was asked to participate was able to provide input into this study; no information was collected about why some individuals declined or why some were unable to participate.

Informants were asked to discuss what they perceived as pathogens of concern to public health in Southern Ontario, the following pathogens were identified: *Salmonella* spp.*, Campylobacter* spp.*, E. coli, Giardia,* Norovirus, *Cryptosporidium*, Hepatitis A, *Listeria*, *S*. Typhi, and Amoebas. Various definitions of importance were considered when ranking pathogens for importance, including: severity/burden of disease, frequency of disease, and/or political pressures to address the disease. Severity of disease was defined as the seriousness of the adverse health effects experienced by infected individuals; frequency of disease was defined as the number of cases due to a particular pathogen; and political pressures were defined in terms of the emphasis placed by organizations to treat and prevent certain enteric infections. The most important pathogens identified were *Salmonella*, *Campylobacter*, *E. coli*, and *Giardia* (Table 1). Although the order was variable, the top four pathogens in this case study were the same as those identified in other Ontario and Canadian reports [1], [4].

**Table 1:** Important pathogens identified

	**Important pathogens identified**												
	**Ranking scores**							**Proportional piling scores**					
**Overall rank**	**Pathogen**	**Interview**			**Focus Group**		**Mean**	**Pathogen**	**Interview**		**Focus group**		**Mean**
**1**	*Salmonella*	5	4	2	5	5	4.2	*Campylobacter*	27	15	21	21.5	21.1
**2**	*Campylobacter*	4	5	3	4	3	3.8	*E. coli*	0	31	30	15.5	19.1
**3**	*E. coli*	1	1	5	1	1	1.8	*Salmonella*	0	14	16	22.5	13.1
**4**	*Giardia*	3	2	1	0	0	1.2	*Giardia*	23	5	12	0	10
**5**	Norovirus	0	0	0	3	3	1.2	*Listeria*	0	0	21	17.5	9.6
**6**	*Cryptosporidium*	2	1	0	2	0	1	Typhoid	36	0	0	0	9
**7**	Hepatitis A	0	0	4	0	0	0.8	Hepatitis A	0	30	0	0	7.5
**8**	*Listeria*	0	0	0	0	3	0.6	Norovirus	0	0	0	24	6
**9**	Typhoid	0	0	0	0	0	0	Amoeba	14	0	0	0	3.5
**10**	Amoeba	0	0	0	0	0	0	*Cryptosproidium*	0	5	0	0	1.3

Using scores obtained from ranking and proportional piling activities, average scores were calculated. Interview and focus group ranks were on a scale of 0 to 5, where 5 was the rank for the most important pathogen. Interview and focus group piling scores were on a scale of 0 to 100, where a higher score was considered a more important pathogen. Using the average rank and piling score, the pathogens were ranked as most important (rank of 1) to least important (rank of 10). The same four pathogens were ranked in the top 4 for both activities.

Once the most important pathogens were identified, informants discussed a wide range of factors that influence the occurrence of these pathogens. A total of 50 contributing factors were identified across all interviews and focus groups. This list was then categorized into nine key themes, which were summarized into three main categories: 1) knowledge and process; 2) risk factors; and 3) susceptibility factors (Table 2). Many informants discussed these themes to varying degrees during both interviews and focus groups. Across all interviews and focus groups (total = 12 discussions), water and food handling themes (risk factors) as well as demographics and behaviour themes (susceptibility factors) were identified and strongly discussed most often (in at least three quarters of discussions). Travel, industry and geography risk factor themes were identified and strongly discussed in less than half of all discussions; public health investigator experience was the only theme strongly discussed under the knowledge and process category. During the discussions, the informants also expanded on the idea that there are many influential factors that affect an individual’s risk of infection and highlighted the complex relationships that exist between the contributing factors and key themes. We have illustrated these complexities in Figure 2.

**Table 2:** Themes and contributing factors identified

	**Themes and contributing factors identified during interviews and focus groups**	
**Category**	**Key theme**	**Contributing factor**
Knowledge and process	Public health experience	▪ How public health experience influences enteric illness investigations (e.g. methodology, education)
	Access to healthcare	▪ Influential factors that affect the availability of healthcare (e.g. remote location, language barrier, under-reporting)
Risk factors	Travel	▪ **International travel** and **domestic travel**
	Food handling	▪ **Food handler**
		▪ **Cross contamination**
		▪ **Temperature control, undercooked food** and **time in storage**
		▪ Food choices: **fresh produce**, **raw milk**, **deli meats**, **soft cheeses**, **sea food**
	Industry	▪ The farm-to-fork continuum: all production stages of food (i.e. **slaughter**, **processing**, **packaging**, **retail**)
	Water	▪ Recreational activities: **swimming, camping, canoeing, hiking**
		▪ Drinking **contaminated surface water** or **well water**
	Geography	▪ **Climate** and **seasonality**
		▪ Spatial factors: **urban versus rural, postal code**
Susceptibility factors	Demographics	▪ Biological factors: **age**, **gender**, **immune-compromised, co-morbidity**
		▪ Work and home environment factors: **occupation**, **socio-economic status**, **living conditions**, **education, university residence, daycare, long-term care facility**
	Behaviours	▪ **Mass gatherings**
		▪ **Person-to-person (e.g. MSM)**
		▪ **Culture:** defined as shared experiences, values, and traditions
		▪ **Ethnicity:** defined as country of origin
		▪ **Rituals/traditions:** food handling practices passed down from generations
		▪ Animal contact: **petting zoos/farms, domestic pets, wildlife reserves**
		▪ Food preferences**: cultural foods**, **traditional foods** (e.g. aboriginal hunting)**, smoked foods**
		▪ **Personal hygiene practices** (e.g. hand-washing)

This table provides a summary of the key themes discussed during the case study. The themes were categorized according to knowledge and process, risk factors, and susceptibility factors. Within these themes, a total of 50 contributing factors (in bold) were identified by informants.

Differences between public health investigators from different health regions were observed in the key themes and contributing factors discussed during semi-structured interviews. For example, informants from the more rural health region (C) identified contributing factors such as raw milk consumption, contaminated well water, and farm animal exposure as important factors affecting enteric disease incidence in their area. In contrast, informants from the very urban health region (B) did not consider the aforementioned rural factors and often highlighted travel in their discussions.

Informants differed in their comfort level when discussing certain subjects. For instance, many informants were hesitant to explore the subject of culture and ethnicity. In one focus group, breaking down and defining the two terms enabled the discussion to continue. While there was consensus that the shared experiences, foods, and values of identified cultures (e.g. work group, university or college students, economic class, religion) act as contributing factors for many enteric pathogens, informants were very hesitant to attribute identified contributing factors (Table 2) to any one ethnicity or cultural group. Informants in this focus group were asked to allocate beans to a matrix of risk factors (e.g. personal hygiene, food preparation, industry, safe water, and travel) and ethnicities (South Asian, Middle Eastern, Chinese, First Nations, and mixed ethnicities); informants allocated more beans for personal hygiene as a risk factor for the mixed ethnicity, an ethnicity most informants self-identified with (Figure 1B).

Informants struggled with future disease trends when asked to predict how disease incidence rates will change over the next 20 years. To help answer this question, one of the focus groups completed a timeline activity where they identified past, present, and future trends for the top pathogens that were previously identified (Figure 1C). The illustration and discussion that took place indicated that many informants did not believe there would be any changes in the rates of enteric disease and assumed that population dynamics would remain the same.

## Discussion

This case study was a successful learning exercise that employed PE techniques in a developed world context. The information collected from each interview and focus group provided a helpful starting point to begin answering our three research questions. The majority of discussions were about contributing factors (risk and susceptibility factors) and the interactions that exist between them. Other information obtained related to observations of comfort levels with certain topics, and the impact of regional differences and public health experience when determining the MLSI.

When asked about important contributing factors for infection with common enteric pathogens, informants identified a multitude of risk and susceptibility factors (Table 2). The factors most commonly discussed fell under the key themes of food handling (e.g. cross contamination, undercooked food, and improper temperature control) and behaviours (e.g. personal hygiene practices). The importance of these contributing factors was consistent with the findings of a previous study that examined public health inspector perceptions of food safety issues [10]. The key theme geography was not discussed in as much detail. A study conducted by Papadopoulos et al. [11] described the same phenomenon when researching risk factors for campylobacteriosis. Informants from that study, as well as the current study, focused on human behaviours and acknowledged that individuals often perceive their food purchases to be safe, and their risk of infection to be low when preparing foods within the home [11]. These findings are consistent with another Ontario report that found over 50% of sporadic enteric cases to be associated with risk behaviours within the home (e.g. unsafe food handling) [12]. The lack of emphasis placed on geography may also have been the result of informants having limited professional experience with contributing factors outside of human behaviour.

Factors contributing to enteric disease often do not act independently, but affect and are affected by many other factors. From the multitude of factors identified by our informants, three complexity diagrams were created to help illustrate some of these associations (Figure 2). The highlighted complexities that exist between the contributing factors and key themes suggests that when study enteric disease there is a need for more holistic approaches that recognise those important interconnections. Similar research supports this concept by acknowledging that enteric disease is a complicated process given the existing interactions and number of ways an individual can acquire an infection [13], [14].

The level of comfort informants had with the topic and the clarity of the questions being asked influenced the relative importance of identified contributing factors. For instance, in one focus group, men having sex with men was introduced as a potential contributing factor but the group quickly dismissed this idea and moved on to list other factors. We hypothesize this was because informants were uncomfortable discussing this subject with their colleagues. However, sexual behaviours such as men having sex with men have been identified as important risk factors for some enteric pathogens (e.g. G*iardia*) [15]. As previously mentioned, informants were also uncomfortable discussing culture and ethnicity. This discomfort was primarily observed in focus group discussions. Informants explained that culture/ethnicity information is not collected by surveillance groups, and therefore felt like they were speculating. For example, one informant stated: “I feel like we’re being prejudicial.” While focus group informants were visibly uncomfortable, the interviewed public health inspectors appeared more open and willing to explore this topic. This may be attributed to the fact that investigators at local health regions have daily exposures to cultural/ethnical differences when investigating enteric disease cases and outbreaks. Group dynamics may have also contributed; semi-structured interviews were conducted with individual investigators whereas in the focus groups informants who stated their opinions and perspectives did so in front of their colleagues.

Informants struggled to explore geography as a theme within the context of Southern Ontario. In one focus group, they expressed that the theme was too limiting given the similar climate and geography across Southern Ontario. While there was uncertainty regarding how deeply this topic was probed, informants went on to explore this theme in terms of a more spatial or environmental context, identifying location clusters or ‘hot spots’ (e.g. hospitals or agricultural centres), seasonality, and differences in contributing factors between urban and rural areas. Overall, interview and focus group informants did not elaborate on the subject of geography, suggesting that additional investigations of environment-related contributing factors may be needed.

In this case study we further considered differences between participating health regions that may impact the presence of certain pathogens. Although informants from semi-structured interviews did not directly address potential differences, observations made across all interviews and focus groups suggested that some dissimilarity exists. While the top pathogens identified (i.e. *Salmonella*, *Campylobacter*, *E. coli*, and *Giardia*) were similar, regional differences were identified and have been reported previously [4]. For example, informants from health region B (urban) commonly identified *S.* Typhi as one of the most concerning pathogens to their region. The high number of *S.* Typhi cases observed in region B was attributed to the large South Asian immigrant population in their region. However, *S.* Typhi was not identified as one of the top important enteric pathogens by inspectors from the other participating health regions or in any of the focus group discussions, suggesting that the distribution of enteric pathogens does vary between regions, although the physical geography may not be the main reason for the observed differences.

Of the top pathogens identified in the case study, three (*Salmonella, Campylobacter*, and *E. coli*) were the same pathogens identified in a previous focus group study in Ontario [10]. These pathogens are the top three leading causes of enteric illness reported in Ontario [12] and are among the top twelve most common domestically acquired enteric pathogens in Canada [1]. The public health inspectors from region B that identified *S.* Typhi as an important pathogen associated it with travel, which is consistent with Canadian estimates that attributed 76% of *S.* Typhi cases to travel [1]. The investigators from region B most often discussed the theme ‘access to healthcare’ and recognised the need for public resources in multiple languages. This need was also acknowledged in a previous study of public health investigators from Central West Ontario [10]. Inspectors from other regions did not identify access to healthcare as an important contributing factor either, perhaps because they did not perceive it as an issue in their region. Under-reporting bias can skew perceptions of common factors that contribute to enteric illness so it is important to acknowledge existing barriers (e.g. language, geographic).

When considering pathogens of concern, informants from both semi-structured interviews and focus groups had varying interpretations of what importance meant to them. In general, informants defined importance as a term that encompassed disease severity and frequency. This may account for the discrepancy between the most important pathogens ranked in the case study and the most frequent pathogens ranked provincially and nationally. We also observed that informants from different public health fields considered different criteria when defining importance. A similar observation was made by Boxstael et al. [16] when they assessed perceptions of food safety issues amongst stakeholders from industry, government, consumer organizations, and universities.

During interviews, public health inspectors explained how infrastructure changes within health regions have been implemented to aid in the determination of the MLSI. Each region has up-to-date manuals, protocols, and pathogen specific standard questionnaires to assist in conducting case follow-up interviews. Investigators also commented that with more experience an investigator develops more confidence and skills to ask more probing questions and form hypotheses. This makes it easier to determine the MLSI. Nonetheless, determining the MLSI is still difficult. One public health investigator explained that while they listen to case stories and form their own probing questions based on responses, “it can be hard to speculate” about the MLSI in many cases. Another inspector noted that they are only able to solve about two out of ten outbreaks. This low solve rate is usually attributed to cases having difficulties recalling what they did or what they ate in weeks past.

When considering low solve rates, it is important to reflect on the complexities that exist between contributing factors. As previously identified, there are many factors, and attributing enteric illness to any one may be unrealistic and simplistic. Identifying and understanding these links and complex relationships can be critical for developing future preventative enteric illness measures. To mitigate this we should consider future changes in MLSI trends and the impact of complex risk factor relationships. However, many informants did not believe any changes would occur and assumed that population dynamics would not change in the future. It is unrealistic to assume that the Canadian population will remain static. Immigration and age trends suggest that in the next few years the median age of Canadians and the number of immigrants to Canada will both increase [17]. Informants also did not consider the emergence of new pathogens or the influence of climate change. Projections suggest that under global warming, Canada will experience longer summers, milder winters, and more extreme precipitation. These climatic changes will affect the risk of enteric illness and subsequently alter disease rates [18]. Informants may not have considered such factors since it can be hard to speculate whether these changes would occur, and subsequently, how they would affect disease incidence; especially if surveillance diagnostics change. One informant did however attribute disease prevalence with new food trends such as food smoking practices. Other informants suggested that the industry needs to implement regulations for such practices.

### Limitations

The case study generated valuable information for the prevention and management of enteric pathogens in Southern Ontario. However, due to the short duration and limited resources of the case study, it should be considered a pilot project. As the data were a product of an inclusive learning exercise that gave everyone the chance to facilitate the PE techniques learned, there were multiple participants collecting information which made data synthesis and analysis difficult. The data presented were also obtained from professional opinions and should therefore only be used as a supplement to existing and future surveillance data. Furthermore, as the focus groups consisted of PHAC and University of Guelph employees at different seniority and expertise levels, individuals may have withheld information if they felt inferior to their colleagues. It is also important to consider group versus one-on-one interview dynamics. The latter allows rapport to be established, where individuals feel more comfortable divulging information compared to group interviews. To overcome these challenges, future studies could conduct follow-up interviews, have informants participate in both an interview and a focus group, or ensure focus groups consist of informants with the same level and type of expertise. Other limitations include the underrepresentation of health regions. Due to limited time, only a small sample of informants was asked to volunteer from three select health regions. Future studies of enteric disease should include informants from local, provincial and federal public health, farmers, processing/packaging workers, retail workers, food safety authorities, scientists, and the general public. Capturing the perceptions of all stakeholders affected by aspects of enteric illness would ensure a more comprehensive analysis and understanding of the most likely source of infection for enteric pathogens across Southern Ontario.

## Conclusions

This study has provided insight into the recognition of important pathogens in Ontario and the complexities that exist for their many contributing factors. These complexities were recognized as issues that affect the occurrence of pathogens, the determination of the most likely source of infection, and the future trends for diseases. Other key observations included 1) a general discomfort among informants when discussing certain contributing factors; 2) a greater emphasis on risk and susceptibility factors that were more associated with human behaviours (e.g. hand washing and food handling) compared to geographic risk factors (e.g. climate and seasonality); and 3) an observed difference in the information shared by informants based on level of expertise, regional location, and (in the focus groups) group composition. The techniques used to collect this information can serve to supplement current and future enteric surveillance and provide a starting point for future studies to explore the identified complexities and ultimately contribute to the development of prevention strategies and policies. Participatory epidemiology afforded this study with a multitude of techniques which are easily adaptable and well-suited for population health studies in a developed and urban setting.

## Abbreviations

AGI: Acute gastrointestinal illness; FoodNet: Canada’s National Integrated Enteric Pathogen Surveillance Program; MLSI: Most likely source of infection; PE: Participatory epidemiology; PHAC: Public Health Agency of Canada.

## Competing interests

The authors declare that they have no competing interests.

## Authors’ contributions

JP and KM developed the workshop and case study proposal and obtained funding for the work. SAH, JP, and KM organized and attended the workshop lectures. KM and JP took additional notes during focus groups. SAH facilitated and took additional notes during interviews and focus groups. SAH prepared the draft for the manuscript. All authors contributed to the interpretation of the results, and have read, reviewed, and approved the final manuscript.

## Pre-publication history

The pre-publication history for this paper can be accessed here:

http://www.biomedcentral.com/1471-2458/14/405/prepub

## Supplementary Material

Additional file 1 Rats Checklist. Click here for file

## References

[B1] Thomas MK Murray R Flockhard L Pintar K Pollari F Fazil A Nesbitt A Marshall B Estimates of the burden of foodborne illness in Canada for 30 specified pathogens and unspecified agents, Circa 2006 Foodborne Pathog Dis 2013 10 7 639 648 10.1089/fpd.2012.1389 23659355 PMC3696931

[B2] Public Health Agency of Canada C-EnterNet: reducing the burden of gastrointestinal disease in Canada 2013 http://www.phac-aspc.gc.ca/c-enternet/overview-apercu-eng.php

[B3] Thomas MK Majowicz SE Pollari F Sockett PN Burden of acute gastrointestinal illness in Canada, 1997–2007 Can Commun Dis Rep 2008 34 5 18800412

[B4] Minsitry of Health and Long-Term Care Ontario annual infectious diseases epidemiology report, 2009 2013 http://www.health.gov.on.ca/en/common/ministry/publications/reports/epi_reports/epi_report_2009.pdf

[B5] Dumoulin D Nesbitt A Marshall B Sittler N Pollari F Informing source attribution of enteric disease: an analysis of public health inspectors’ opinions on the “most likely source of infection.” Environ Health Rev 2012 55 27 36

[B6] Majowics SE Edge VL Fazil A McNab WB Dore KA Sockett PN Flint JA Middleton D McEwen SA Wilson JB Estimating the under-reporting rate for infectious gastrointestinal illness in Ontario Can J Public Health 2005 96 3 178 181 15913079 10.1007/BF03403685 PMC6975884

[B7] Region of Waterloo Public Health Waterloo region status report – enteric disease 2005–2009 2013 http://chd.region.waterloo.on.ca/en/researchResourcesPublications/resources/EntericDiseaseStatus.pdf

[B8] Food and Agricultural Organization of the United Nations Manual on participatory epidemiology: methods for collection of action-oriented epidemiological intelligence 2013 http://www.fao.org/docrep/003/x8833e/x8833e00.HTM

[B9] Green JC Caracelli VJ Graham WF Toward a conceptual framework for mixed methods evaluation designs Educ Eval Policy Anal 1989 11 255 274 10.3102/01623737011003255

[B10] Pham MT Jones AQ Sargeant JM Marshall BJ Dewey CE A qualitative exploration of the perceptions and information needs of public health inspectors responsible for food safety BMC Public Health 2010 10 345 10.1186/1471-2458-10-345 20553592 PMC2905330

[B11] Papadopoulos A Vellekoop E Pham M Young I Britten N Using risk factor weighting to target and create effective public health policy for camplylobacteriosis prevention in Ontario, Canada Am J Public Health Res 2013 1 32 37

[B12] Lee MB Middleton D Enteric illness in Ontario, Canada, from 1997 to 2001 J Food Prot 2003 66 953 961 12800994 10.4315/0362-028x-66.6.953

[B13] Varga C Pearl DL McEwen SA Sargeant JM Pollari F Guerin MT Incidence, distribution, seasonality, and demographic risk factors of Salmonella Enteritidis human infections in Ontario, Canada, 2007–2009 BMC Infect Dis 2013 13 212 10.1186/1471-2334-13-212 23663256 PMC3655886

[B14] Parmley EJ Pintar K Majowicz S Avery B Cook A Jokinen C Gannon V Lapen DR Topp E Edge TA Gilmour M Pollari F Reid Smith R Irwin R A Canadian application of One Health: integration of Salmonella data from various Canadian surveillance programs (2005–2010) Foodborne Pathog Dis 2013 in press 10.1089/fpd.2012.1438 23786604

[B15] Public Health agency of Canada Pathogen safety data sheets and risk assessment 2013 http://www.phac-aspc.gc.ca/lab-bio/res/psds-ftss/index-eng.php

[B16] Boxstael SV Habib I Jacxsens L Vocht MD Baert L Van De Perre E Rajkovic A Lopez-Galvez F Sampres I Spanoghe P De Meulenaer B Uyttendaele M Food safety issues in fresh produce: bacterial pathogens, viruses and pesticide residues indicated as major concerns by stakeholders in the fresh produce chain Food Control 2013 32 190 197 10.1016/j.foodcont.2012.11.038

[B17] Statistics Canada Some facts about the demographic and ethnocultural composition of the population 2013 http://www.statcan.gc.ca/pub/91-003-x/2007001/4129904-eng.htm

[B18] Charron DF Thomas MK Waltner-Teows D Aramini JJ Edge T Kent RA Maarouf AR Wilson J Vulnerability of waterborne diseases to climate change in Canada: a review J Toxicol Environ Health, Part A 2004 67 1667 1677 10.1080/15287390490492313 15371208

