# Supplementary material for: Using participatory epidemiology to assess factors contributing to common enteric pathogens in Ontario: results from a workshop held at the Ontario Veterinary College, University of Guelph, Ontario
Source: BMC Public Health. 2014 Apr 27;14:405. doi: 10.1186/1471-2458-14-405 (PMC4032165; doi:10.1186/1471-2458-14-405)
Supplement: Additional file 1 — Rats Checklist. [file 1471-2458-14-405-S1.docx]

| **RATS CHECKLIST** | | | |
| --- | --- | --- | --- |
| **ASK THIS OF THE MANUSCRIPT** | **THIS SHOULD BE INCLUDED IN THE MANUSCRIPT** | **INCLUDED**  **(X)** | **JUSTIFICATION** |
| **R Relevance of study question** |  |  |  |
| Is the research question interesting?  Is the research question relevant to clinical practice, public health, or policy? | Research question explicitly stated  Research question justified and linked to the existing knowledge base (empirical research, theory, policy) | X | Goals and research question stated in background section  Supported by literature in background section |
| **A Appropriateness of qualitative method** |  |  |  |
| Is qualitative methodology the best approach for the study aims?   - *Interviews:* experience, perceptions, behaviour, practice, process - *Focus groups:* group dynamics, convenience, non-sensitive topics - *Ethnography:* culture, organizational behaviour, interaction - *Textual analysis:* documents, art, representations, conversations | Study design described and justified i.e., why was a particular method (e.g., interviews) chosen? | X | Study design described in methodology  For more detail we provided an example of one of the preliminary questions (p. 7)  Purpose of workshop was to use participatory epidemiology methodology and evaluate its use in the developed world context |
| **T Transparency of procedures**  *Sampling* |  |  |  |
| Are the participants selected the most appropriate to provide access to the type of knowledge sought by the study?  Is the sampling strategy appropriate? | Criteria for selecting the study sample justified and explained   - *theoretical:* based on preconceived or emergent theory - *purposive:* diversity of opinion - *volunteer:* feasibility, hard-to-reach groups | X | Convenience sample taken  Explained in limitations section |
| *Recruitment* |  |  |  |
| Was recruitment conducted using appropriate methods? | Details of how recruitment was conducted and by whom | X | Details provided in methodology |
| Is the sampling strategy appropriate? |  | X | A convenience sample was chosen as this study was considered to be a pilot project  Discussed in limitations section |
| Could there be selection bias? | Details of who chose not to participate and why | X | Bias discussed in limitations section  We provided more detail about participation (end of p. 9) |
| *Data collection* |  |  |  |
| Was collection of data systematic and comprehensive? | Method(s) outlined and examples given (e.g., interview questions) | X | Described in methodology |
| Are characteristics of the study group and setting clear? | Study group and setting clearly described | X | We further clarified where the interviews and focus groups were held (p. 7) |
| Why and when data collection was stopped, and is this reasonable? | End of data collection justified and described | X | Study was done as part of 2 week workshop and pilot study |
| *Role of researchers* |  |  |  |
| Is the researcher(s) appropriate? How might they bias (good and bad) the conduct of the study and results? | Do the researchers occupy dual roles (clinician and researcher)? Are the ethics of this discussed? Do the researcher(s) critically examine their own influence on the formulation of the research question, data collection, and interpretation? | X | No dual roles  Ethics discussed in methodology  As described in methodology, the research question was formulated ahead of the workshop, data collection and analysis was done as a group |
| *Ethics* |  |  |  |
| Was informed consent sought and granted? | Informed consent process explicitly and clearly detailed | X | Described in methodology |
| Were participants’ anonymity and confidentiality ensured? | Anonymity and confidentiality discussed | X | Described in methodology  An additional sentence was added to describe the information sheet given to informants prior to an interview or focus group (p. 6) |
| Was approval from an appropriate ethics committee received? | Ethics approval cited | X | Not required as we were obtaining professional opinions |
| S Soundness of interpretive approach Analysis |  |  |  |
| Is the type of analysis appropriate for the type of study?   - thematic: exploratory, descriptive, hypothesis generating - framework: e.g., policy - constant comparison/grounded theory: theory generating, analytical   Are the interpretations clearly presented and adequately supported by the evidence? | Analytic approach described in depth and justified  Indicators of quality: Description of how themes were derived from the data (inductive or deductive)  Evidence of alternative explanations being sought  Analysis and presentation of negative or deviant cases | X | Described in methodology |
| Are quotes used and are these appropriate and effective? | Description of the basis on which quotes were chosen  Semi-quantification when appropriate  Illumination of context and/or meaning, richly detailed | X | 1 quote was used in the discussion section as an example to emphasize the level of discomfort experienced in a focus group  We did not provided further detail about why this quote was chosen |
| Was trustworthiness/reliability of the data and interpretations checked? | Method of reliability check described and justified e.g., was an audit trail, triangulation, or member checking employed? Did an independent analyst review data and contest themes? How were disagreements resolved? | X | Triangulation of data (described in methodology) |
| Discussion and presentation |  |  |  |
| Are findings sufficiently grounded in a theoretical or conceptual framework?  Is adequate account taken of previous knowledge and how the findings add? | Findings presented with reference to existing theoretical and empirical literature, and how they contribute | X | Literature cited in background and discussion sections |
| Are the limitations thoughtfully considered? | Strengths and limitations explicitly described and discussed | x | Described in limitations section |
| Is the manuscript well written and accessible? | Evidence of following guidelines (format, word count)  Detail of methods or additional quotes contained in appendix  Written for a health sciences audience | X | Guidelines followed  Statement added about adherence to RATS checklist (p. 9) |
| Are red flags present? These are common features of ill-conceived or poorly executed qualitative studies, are a cause for concern, and must be viewed critically. They might be fatal flaws, or they may result from lack of detail or clarity. | Grounded theory: not a simple content analysis but a complex, sociological, theory generating approach  Jargon: descriptions that are trite, pat or jargon filled should be viewed sceptically  Over interpretation: interpretation must be grounded in "accounts" and semi-quantified if possible or appropriate  Seems anecdotal, self-evident: may be a superficial analysis, not rooted in conceptual framework or linked to previous knowledge, and lacking depth  Consent process thinly discussed: may not have met ethics requirements  Doctor-researcher: consider the ethical implications for patients and the bias in data collection and interpretation | X | We avoided use of jargon  Analysis was based on the conceptual framework of participatory epidemiology |
